# shRNA mediated knockdown of Nav1.7 in rat dorsal root ganglion attenuates pain following burn injury

**DOI:** 10.1186/s12871-016-0215-0

**Published:** 2016-08-11

**Authors:** Weihua Cai, Jing Cao, Xiuhua Ren, Liang Qiao, Xuemei Chen, Ming Li, Weidong Zang

**Affiliations:** 1 Department of Anatomy, School of Basic Medical Sciences, Zhengzhou University, Henan, China; 2 Department of E.N.T, Zhoukou Central Hospital, Henan, China

**Keywords:** Nav1.7, Burn injury pain, Lentiviral vector, shRNA, Dorsal root ganglion

## Abstract

**Background:**

Abnormal acute pain after burn injury still torments patients severely. In this study, we investigated that one voltage gated sodium channel Nav1.7 plays a vital role in lowering heat pain threshold after burn injury, and the hypothesis that knockdown of Nav1.7 attenuates pain following burn injury.

**Methods:**

Sixty eight adult male Sprague–Dawley rats were divided into 4 treatment groups: (1) sham, which hind paw was put on the room temperature metal plate for 15 s (2) burn model, which hind paw was put on the 85 °C metal plate for 15 s. (3) Burn injury + lentiviral vector -SCN9AsiRNA-GFP (LV- SCN9AsiRNA-GFP group, *n* = 18), which receive the DRG microinjection of LV- SCN9AsiRNA-GFP on the zero day. (4) Burn injury + lentiviral vector negative control (LV-NC-GFP group, *n* = 18), which receive the DRG microinjection of empty lentiviral vector on the zero day.

**Results:**

Both mechanical and heat threshold were measured from day 1 to 21. Meanwhile, expression of sodium channels Nav1.7 in injured dorsal root ganglia were measured on post-operative days 7(POD 7). Rats exhibited decreased thresholds on both mechanical allodynia and thermal withdrawl latency, accompanied by increased Nav1.7 and c-fos expression in dorsal root ganglion (DRG). And knockdown of Nav1.7 in L5DRG led to the attenuation of burn injury-induced mechanical allodynia and thermal hyperalgesia in the rats.

**Conclusion:**

We provide evidence that shRNA mediated knockdown of Nav1.7 attenuates burn induced pain in rats as well as decreased the activiation of c-fos protein.

## Background

Burn injury occurs with a high prevalence and causes high morbidity [12]. Globally, nearly 11 million people have burn pain which is severe enough to require medical attention, each year [17]. Burn pain is a spontaneous ongoing unpleasant feeling, and induces both persistent thermal and mechanical hyperalgesia [19]. Currently, the cure for burn-injury remains a challenge. Therefore, great effects have focused on understanding basic mechanisms of pain caused by burn injury [3, 19].

Voltage gated sodium (Nav) channels are known to play a key role in the induction of nociceptors, especially in the initial rising phase of the action potential. Nav channels are composed of a family of α-subunit (Na v 1.1–1.9) and one or more β-subunits (b1-b4) [2, 13]. Up to now, 9 subtypes of Nav channels have been identified in human, and changes in their expression may underlie hypersensitivity in pain states [5]. Among Nav channels, Nav1.7 is encoded by the gene SCN9A [26]. Nav1.7 has raised interest among the pain researchers, since it has been found to be related to paroxysmal extreme pain disorder [15], erythromelalgia [4], and painful neuropathy in type 2 diabetes [7]. Recently, Nav1.7 also has been found to be essential for lowering heat pain threshold after burn injury [19]. Therefore, Nav1.7 is considered to play a critical role in pain pathways. In addition, Nav1.7 channel availability sets the AP shape, initiation of burst firing [24] In this study, we aimed to investigate the role of Nav1.7 in burn injury.

In this study, we chose lentivirus vector to deliver Nav1.7 shRNA into dorsal root ganglion (DRG) neurons to knockdown SCN9A gene to investigate the role of Nav1.7 in rat model of burn injury. In addition, we examined the expression of c-fos protein because c-fos overexpression was associated with high-grade lesion [6, 16], and the c-fos has been considered as a rapidly expressed gene marker of pain [9] or nociceptive neuronal activation [10]. Small hairpin RNA (shRNA) is a new tool for gene knockdown. For the expression of shRNA in the cells it is usually delivered into cells by plasmids or viral or bacterial vectors [8, 23]. Slow virus vector can not only transfect undivided neurons, but also can be integrated into the host genome, leading to the silencing of target genes for a long time and even permanently [11, 20]. Recently, the most common carrier system for the expression of shRNA is to transfect viral vector harboring the promoter of RNA pol III as well as a piece of special structure of its downstream gene into the host cell, shRNA could be cut into siRNA by Dicer enzyme in the cells [18, 22, 27].

## Methods

### Burn injury model

Animal experiments were approved by the Ethics committee of Henan province. Zhengzhou, Henan, China.

Male SD rats (weight 180–220 g) were purchased from Animal Experiment Center of Henan Province, housed 4 per cage and maintained on a 12 h light/dark schedule in a temperature-controlled at 25 °C environment with available access to food and tap water. The rats were randomly divided into 2 groups (*n* = 16): Burn injury group: A metal plate cover floated on which water circulated from 85 °C water bath was arranged. One hind paw of each rat was held in contact with the metal plate cover for 15 s to establish the burn model. Sham group: Sham-treated animals went through the identical treatment except that the hind paw was placed on the room temperature metal plate instead of 85 °C. Burn injury was performed n the rats as described previously [19]. Anesthesia was induced with 5 % isoflurane and maintained at 2.5 % (V/VO_2_ 2 l.^min-1^) before the process and remained throughout the duration of the process. Rats were allowed to recover from anesthesia in their home cages within 10 min after operation. According to the behavioral test results, animals displayed the extreme acute pain on 7 d after operation. Therefore, animals were sacrificed on 7 d to collect DRGs.

### Behavioral test

Rats were habituated to the test apparatus for at least 30 min before test on test days.

Behavioral test was performed blindly to treatment group. Hind paw radiant heat (Hargreaves’) test: Rats were placed in plastic chambers on a glass surface maintained at 25 °C. A radiant heat source was focused on the hind paw and latency to respond was recorded in three trials per paw, separated by at least 10 min. Heat intensity was 30 % and cutoff to avoid tissue damage was 30 s. von Frey test of mechanical threshold: rats were placed in plastic chambers on a wire mesh grid and stimulated with von Frey filaments according to the up-down method described previously [1].

### Application of lentiviral vector-mediated shRNA

Thirty six male SD rats were randomly divided into 2 groups (*n* = 18): Burn injury + empty lentiviral vector -SCN9AsiRNA-GFP (LV3- SCN9AsiRNA-GFP group), burn injury + lentiviral vector negative control (LV3-NC-GFP group). Recombinant Lentivirus were offered by Shanghai GenePharma Co.,Ltd. On the zero day, burn injury model rats in LV3-NC group and LV3- SCN9A group received DRG microinjection [14] of 4 μl empty lentiviral vector and LV3- SCN9A vector, respectively. On the day before burn injury and 4 d, 7 d, 14 d, 21 d after burn injury (Post Operation Days, POD4, POD7.POD14, and POD21), mechanical withdrawal threshold and thermal withdrawal latency were measured. Rats were scarified on 7 d after operation and DRG was collected.

### Immunofluorescence

Under deep anesthesia with isoflurane, the rats were perfused with normal saline followed by cooled 4 % paraformaldehyde in 1 M phosphate buffer. L4 -6 DRGs were collected at 4 d, 7 d, 14 d, and 21 d. DRG was paraffin embedded and sectioned at 20 mm. For single labeling, 3 sets of DRG sections (4–5 sections/DRG) were collected. After being dewaxed by gradient with xylene ethanol, the sections were rinsed by PBS 5 min/times. After Antigen repair, the sections were blocked with goat serum for 2 h at room temperature. Then, the sections were incubated with Nav1.7 mouse monoclonal antibody (1 mg/mL; abcam) and C-fos goat anti-mouse antibody (1:500; Beijing Zhongshan biotechnology) for 30 min at 37 °C then at 4 °C overnight. The sections were then incubated with goat anti-rabbit antibody conjugated with FITC (1:80; Shanghai Weiao biotech) or donkey anti-mouse antibody conjugated with Cy3 (1:200; Jackson ImmunoResearch) for 2 h or DAPI (1:1000; Sigma) at room temperature (RT) and covered with BSA (10 %; Shanghai Weiao biotech). All stained sections were viewed with an epifluorescence microscope (Olympus Corporation, Japan).

### Western blot analysis

Total protein was extracted from L4–6 DRGs of the rats using tissue protein extraction reagent (Weiao Biotech, Shanghai, China). The proteins were loaded and separated by sodium dodcyl sulfate (SDS)- polyacrylamide gels, electrophoretically transferred onto polyvinylidene fluoride membranes (Millipore, MA, US). The membranes were then blocked with the blocking buffer (5 % fat free dry milk with TBST) for 2 h at RT and incubated with rabbit anti-Nav1.7 (1:600, Abcam, England) at 4 °C for 2 nights. Finally, the membranes were incubated with goat anti-rabbit antibody (1:800, Zhongshan Jinqiao, China) for 2 h at RT and signal was detected with ECL detection reagents (Alphalmager proteinsimple, San Jose, USA).

### Statistical analysis

For statistical analysis, GraphPad Prism software was used. Behavioral data and immune fluorescence intensity were analyzed by either the Student’s t-test to compare 2 groups or ANOVA followed by planned comparisons of multiple groups. In both cases, when significant main effects were observed, *P* < 0.05 was considered to be statistically significant.

## Results

### Nociceptive behaviors after burn injury

After burn injury, SD rats displayed increased sensitivity to heat and mechanical stimuli. To elucidate basic mechanism of burn injury-induced sensitization, we first established a model of focal second-degree burn in rats.

**Fig. 1:**
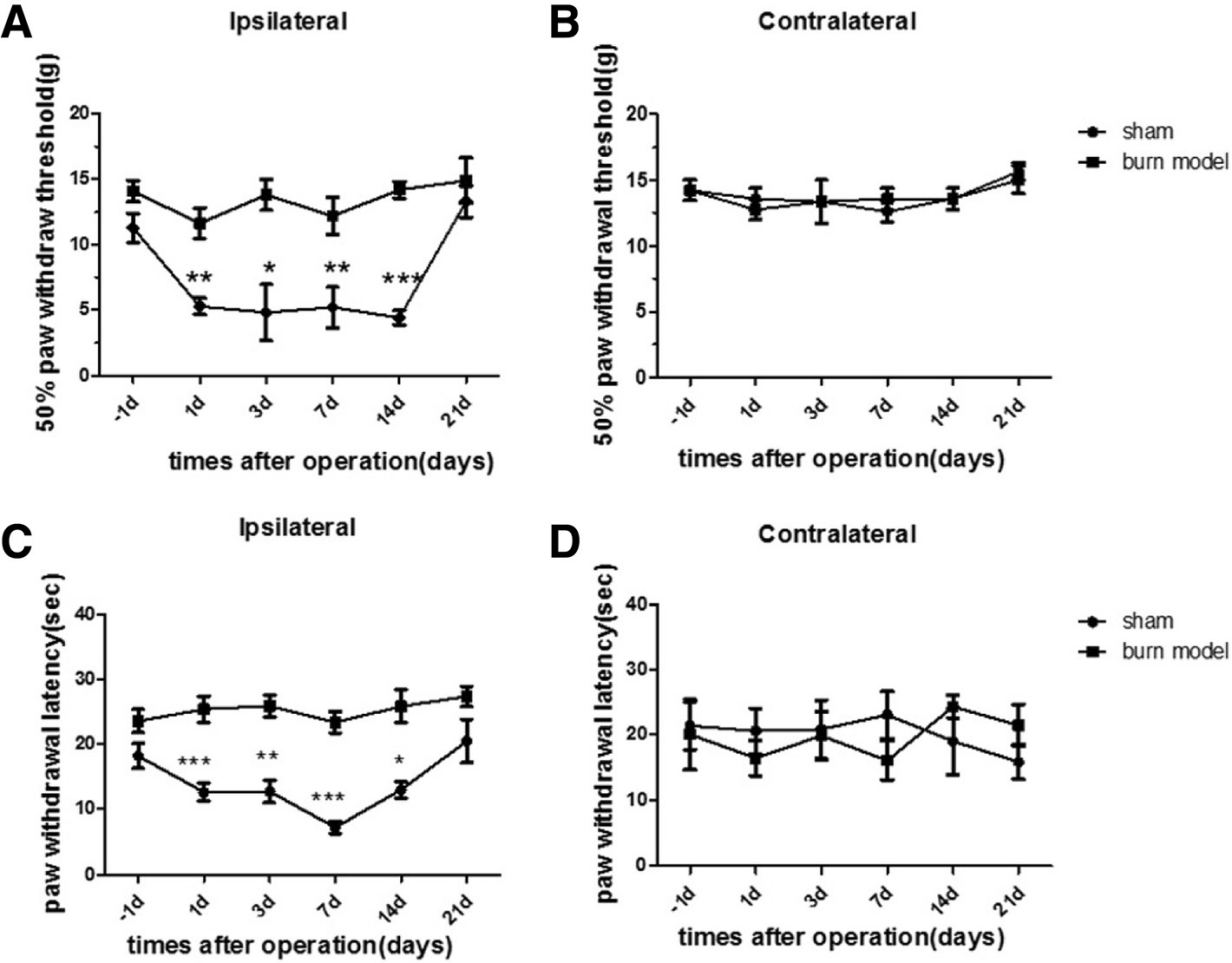
Bury injury causes the hypersensitivity to heat and mechanical stimuli in rat. (**a**) Response thresholds to mechanical stimuli in the von Frey test are strongly decreased on the ipsilateral side in burn model animals and unchanged in sham-treated animals. (**b**) neither on the contralateral side. Mechanical hypersensitivity resolves within 3 weeks. (*p < 0.05, **p < 0.01, ***p < 0.001, NS: not statistically significant; n =16 rats/group.) (**c**) Response thresholds to radiant heat stimuli (Hargreaves’test) are strongly decreased on the ipsilateral side in burn model animals and unchanged in sham-treated animals. (**d**) And there was no change on the contralateral side. Heat hypersensitivity is present at the earliest time point assessed and resolves within 3 weeks. (*p < 0.05, **p < 0.01, ***p < 0.001vs sham group; n=16 rats/ group.)

We found that withdrawal thresholds to heat and mechanical stimuli were reduced in the burned hind paw at the earliest time point examined (2–4 h after the heat pain) and the decreases lasted 14–21 days (Fig. 1a, b). However, no obvious changes in response thresholds were observed in sham-treated rats or in the contralateral hind paw of burn model rats.

### Protein expression change after burn injury

**Fig. 2:**
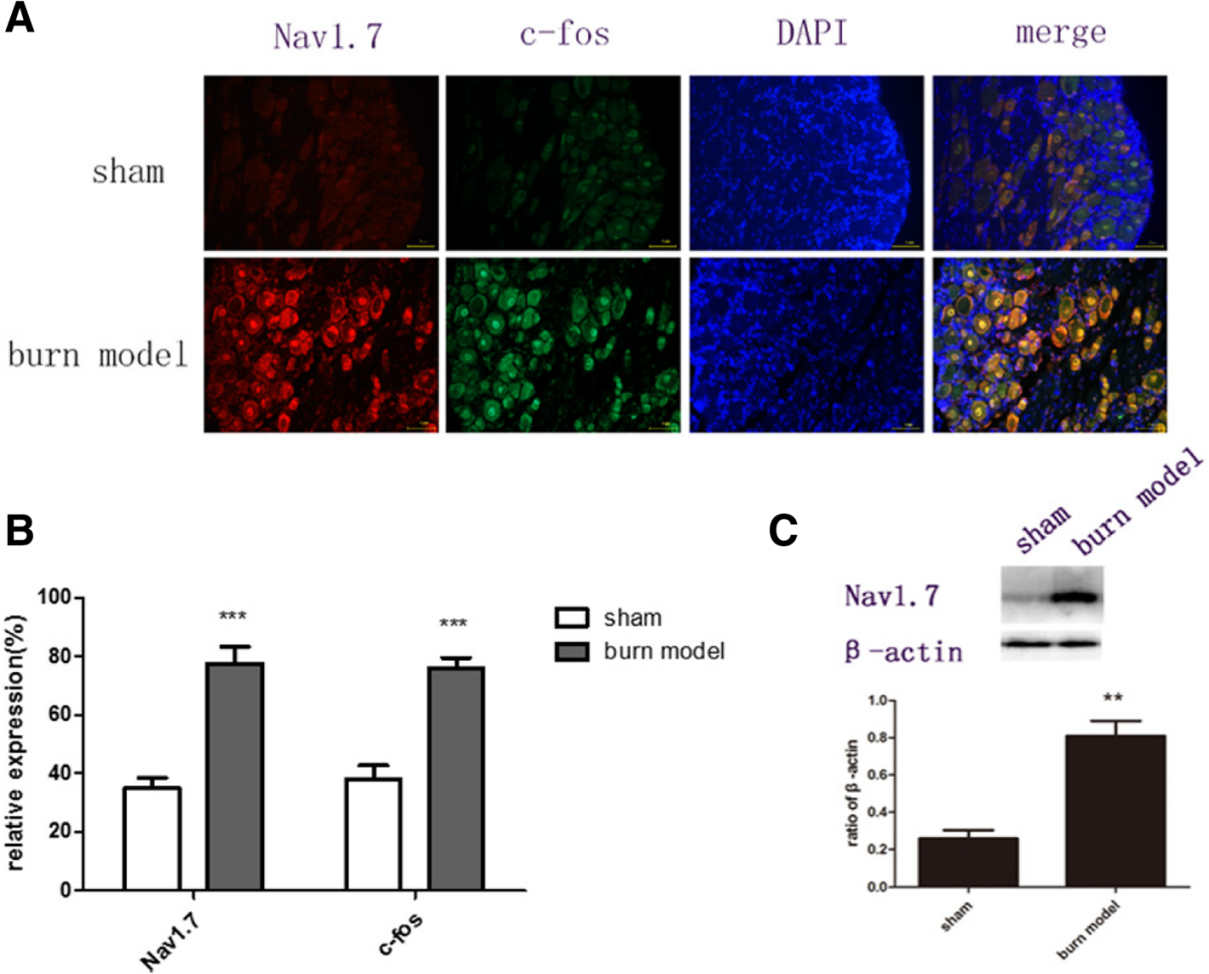
Effect of burn injury on number of Nav1.7 and c-fos positive neurons, andexpression of Nav1.7 protein 7 d after burn injury in DRGs. **a, b** Nav1.7 and c-fos labeled neurons were significantly increased after burn injury. **c** The expression of Nav1.7 protein was up-regulated in the DRGs of burn model rats. (*p < 0.05, **p < 0.01, ***P < 0.001 versus the sham group, NS: not statistically significant; *n* = 3/group)

Next we examined the expression of Nav1.7 and c-fos protein in DRG neurons of burn injury rat model. Immunohistochemical and Western blot analysis indicated that Nav1.7 expression and nerve injury marker c-fos protein expression were increased in DRG neurons of burn injury rats on day 7 after injury (Fig. 2). These data indicate that the expression of Nav1.7 as well as c-fos protein was related to burn injury.

### Nociceptive behaviors after treatment

**Fig. 3:**
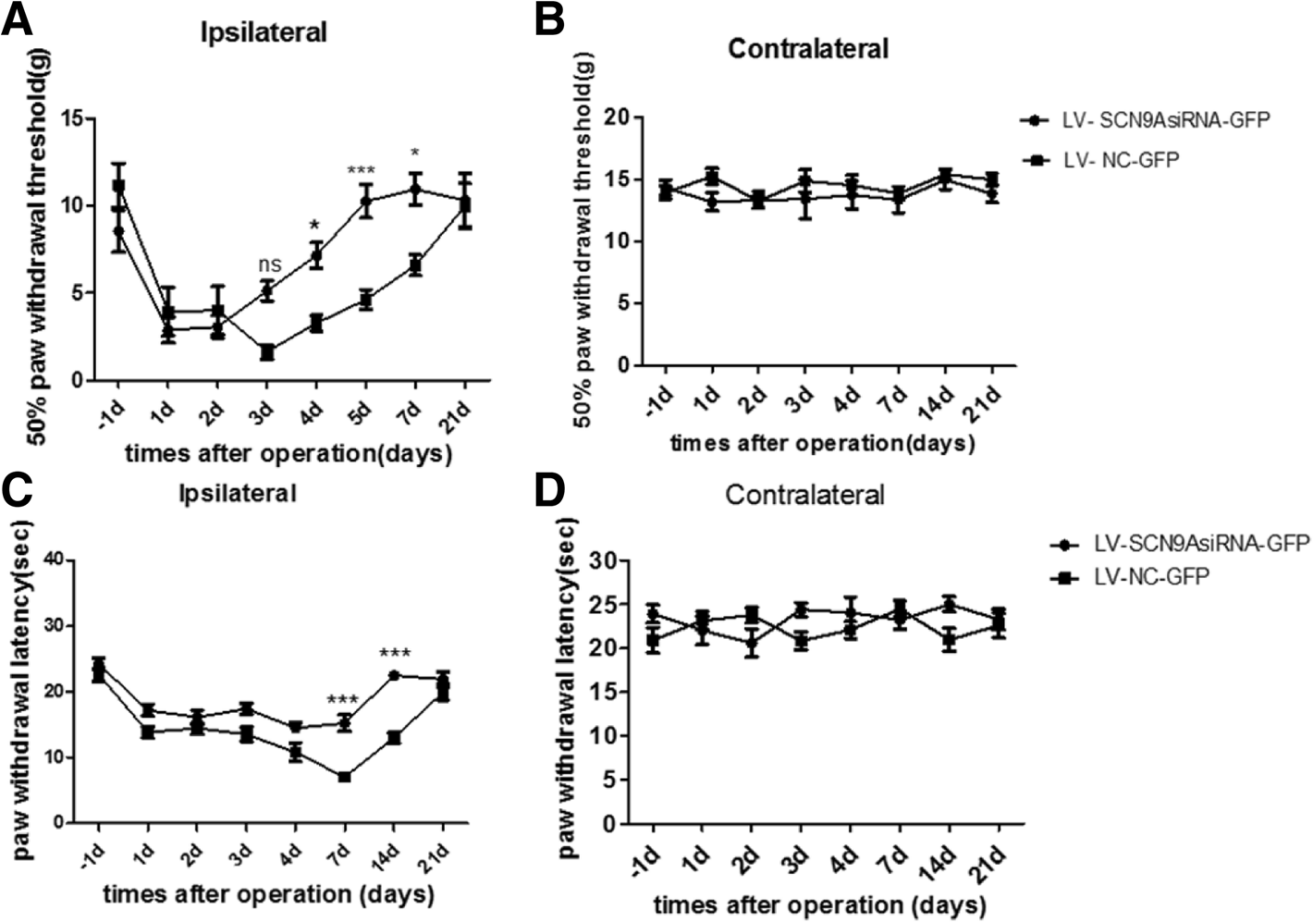
The change of hypersensitivity to heat and mechanical stimuli on burn injury model of rats after injected with LV-SCN9AsiRNA-GFP and LV-NC-GFP. (**a**) Response thresholds to mechanical stimuli in the von Frey test are strongly increased after treated with LV-SCN9AsiRNA-GFP and unchanged in LV-NC-GFP-treated animals. (**b**) And there was no difference of mechanical hypersensitivity between two groups on the contralateral side. (*p < 0.05, **p < 0.01, ***P <0.001, NS: not statistically significant; n =18 rats/group). (**c**) Response thresholds to radiant heat stimuli (Hargreaves’ test) are obviously revived on the ipsilateral side after treated with LV-SCN9AsiRNA-GFP while LV-NC-GFP didn't. (**d**) Heat hypersensitivity has no difference between two groups on the contralateral side. (*p < 0.05, **p < 0.01, ***P <0.001, NS: not statistically significant; versus the LV-NC-GFP group; n =18 rats/group.)

To knockdown Nav1.7 efficiently after burn injury in adult rats, we injected lentiviral vector harboring Nav1.7 shRNA into L4 DRG which innervates partial area of the hindpaw. Lentiviral vector-mediated shRNA on 0d after burn injury inhibited the development of burn injury pain, attenuated both withdrawal thresholds to heat and mechanical stimuli on 21 d after burn injury (Fig. 3). Furthermore, mechanical withdrawal threshold was alleviated from 3 d after burn injury (*P* = 0.0008) while heat threshold was increased from 7 d after burn injury (*P* < 0.0001).

### Protein expression change after treatment

**Fig. 4:**
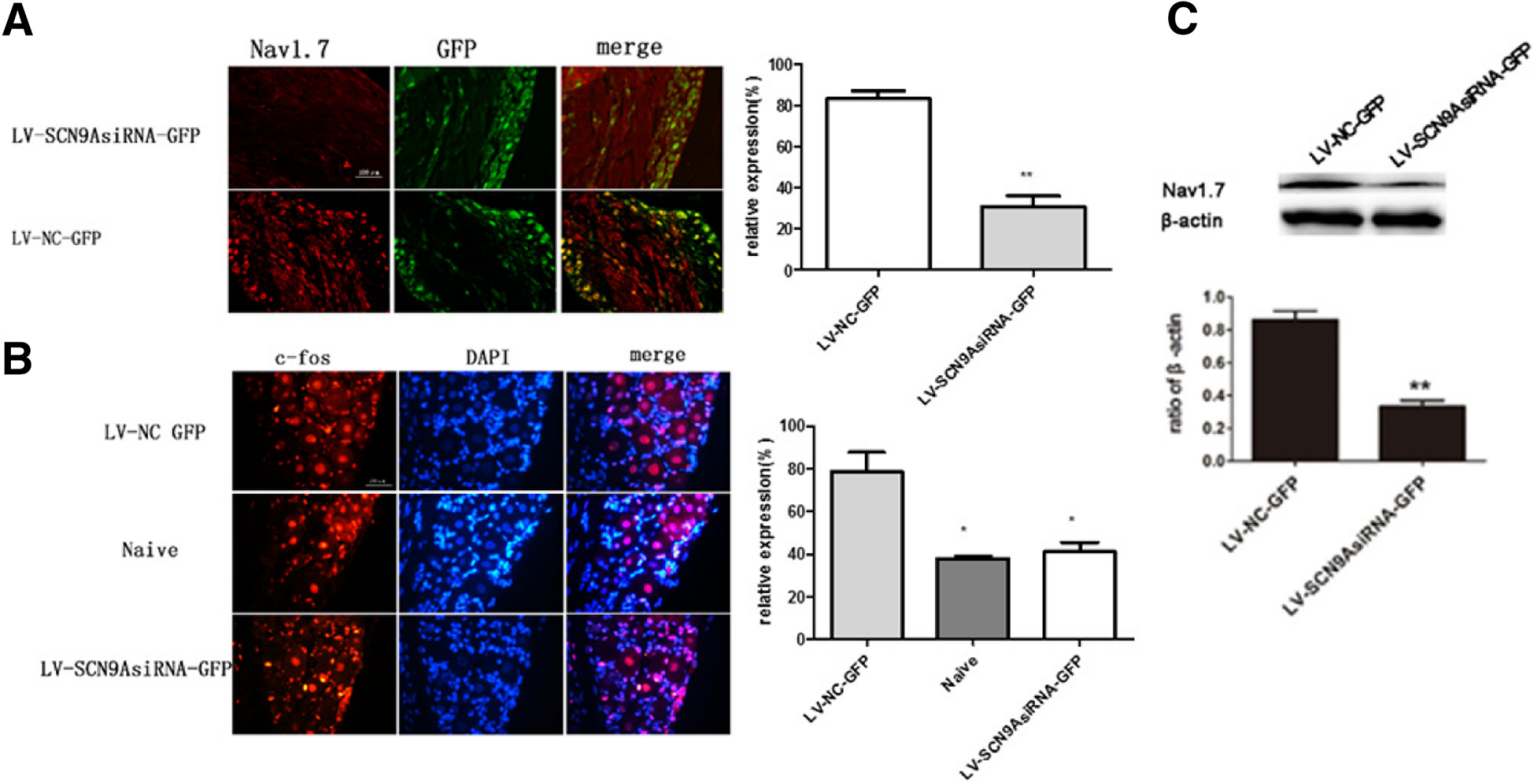
Effects of siSCN9A on the burn -induced increase in Nav1.7 expression in the ipsilateral DRGs on day 7 after burn injury. **a** The amount of Nav1.7 reduced in DRG after burn injury while the GFP remained the same after administration to knockdown SCN9A gene. **b** the increased c-fos positive neurons have been reduced slightly after the SCN9A was knocked down; **c** and the up-regulated expression of Nav 1.7 was attenuated in injured DRGs after SCN9A was knocked down. (*p < 0.05, **p < 0.01, ***P < 0.001, NS: not statistically significant; versus the LV-NC-GFP group; *n* = 3/group)

To further examine the effects of Nav1.7 knockdown on the expression of Nav1.7 and c-fos protein in DRG neurons, we performed immunohistochemical and Western blot analysis. Compared with negative negative control group, Nav1.7 expression was significantly reduced in the DRG of the rats in LV- SCN9A group on 7 d after burn injury. And the burn injury didn’t occurred the significant increase on c-fos protein in the DRG of the rats in LV- SCN9A group on 7 d after burn injury, while the negative control group did (Fig. 4).

## Discussion

Burn injury arouses worldwide attention due to the large number of the patients and the abnormal acute pain which torments the patients in duration of therapy. Burn injury causes the release of various inflammatory mediators, as well as the Nav1.7 and c-fos proteins which are related to local and systemic inflammation or neurons activation. In this study, we established a rat model of focal second-degree burn injury to simulate the burn condition, for the investigation of mechanisms underlying burn-related pain. Using this model, we performed behavioral experiments and also examined the expression of sodium channel isoforms in the peripheral nervous system. We found that Nav1.7 is associated with burn-induced hypersensitivity to heat stimuli. Next we injected lentiviral vector-mediated shRNA into DRG microinjection to knockdown SCN9A gene to understand the role of Nav1.7 in burn injury induced pain.

Recently, several Nav1.7 inhibitor has been utilized, for instance,benzazepinones could block Nav1.7 sodium channels, and methadone could be a local anaesthetic-like inhibitor of Nav1.7 sodium channels [21]. However, the application of these inhibitors has several concerns, including the specificity and side effects such as neurotoxicity. Therefore, in our present study, we chose the lentiviral vector-mediated shRNA to specifically knockdown Nav1.7 to elucidate the role of Nav1.7 in burn injury pain.

To our knowledge, there are various ways to deliver drugs and biological to DRG, including epidural injection (Long term catheterization), intrathecal injection [25], direct injection of the ganglion (DRG microinjection), and injection of peripheral tissue or the peripheral nerve like sciatic nerve. Considering that Nav1.7 is mostly expressed in DRG, we chose the DRG microinjection method to deliver lentiviral vector shRNA.

Previous study reported that the expression of Nav1.7 was increased in DRG neurons of burn-injury rats, thus we hypothesized that inhibiting Nav1.7 expression could prevent or relieve pain after burn injury, and alleviate the lesion in the burn injury-related neurons by reducing the expression of c-fos protein. In this study, we injected lent virus vector Nav1.7 shRNA to the DRG of the burn-injury rats at the day we perform the burn injury. Our data showed that the MWT of the left hind leg of burn-injury rat model had a significant rise at 4 d, 7 d and 21 d, exhibiting an apparent resistance to damage effect. We chose the 7 d after burn injury as the typical time point for protein expression analysis by Western Blot, because it’s the time point that the ipsilateral withdrawal thresholds and latencies in the shRNA groups were both significantly increased.

## Conclusions

In summary, our data showed that shRNA mediated knockdown of Nav1.7 in L5DRG led to the attenuation of burn injury-induced mechanical allodynia and thermal hyperalgesia as well as the decrease of the activation of c-fos protein.in the rat hind paw. Therefore, Nav1.7 is a potential target to treat burn injury pain, and lentivirus mediated Nav1.7 shRNA have potential clinical application for gene therapy of burn injury pain.

## Abbreviations

DRG, dorsal root ganglion; LV, lentiviral vector; NC, negative control; POD, post-operative days; PWL, paw withdrawal latency; PWT, 50 % paw withdrawal threshold; RT, room temperature
